# The Interaction Between Cognitive Abilities and White Matter Hyperintensity Phenotypes: A Novel Perspective on Bidirectional Causality

**DOI:** 10.1002/brb3.70313

**Published:** 2025-02-09

**Authors:** Xiaolu Ren, Ting Li, Fang Li, Shan Liu

**Affiliations:** ^1^ Department of Radiology General Hospital of Ningxia Medical University Yinchuan China; ^2^ School of Health Sciences Universiti Sains Malaysia Kelantan Malaysia; ^3^ Department of Neurology General Hospital of Ningxia Medical University Yinchuan China

**Keywords:** bidirectional, causal relationship, cognitive function, Mendelian randomization, white matter, hyperintensity volume

## Abstract

**Purpose:**

This study utilizes bidirectional Mendelian randomization (MR) to examine the causal relationships between white matter hyperintensity (WMH) phenotypes—namely, WMH volume, fractional anisotropy (FA), and mean diffusivity (MD)—and cognitive abilities, including cognitive performance, intelligence, and overall cognitive function.

**Methods:**

This study leverages genetic variation data from genome‐wide association study (GWAS) datasets and employs a bidirectional two‐sample MR analysis. The analysis incorporates MR Egger, weighted median, weighted mode, and inverse variance weighted (IVW) methods to assess the bidirectional causal relationship between cognitive abilities and WMH volume, FA, and MD.

**Results:**

This study employed MR to explore the causal relationships between WMH volume, FA, MD, and cognitive outcomes. Most MR methods yielded nonsignificant *p* values (>0.05) and wide confidence intervals. Heterogeneity tests indicated no significant heterogeneity or pleiotropy between WMH volume and cognitive performance or intelligence. However, significant heterogeneity was found between WMH volume and cognitive function, FA with cognitive performance and intelligence, and MD with cognitive performance and intelligence. Reverse analysis also revealed no significant causal relationships.

**Conclusions:**

This study suggests that the bidirectional causal effects between cognitive abilities and WMH volume, FA, and MD are minimal or nonsignificant and highlights data heterogeneity as a concern.

## Graphical abstract

The study uses a two‐sample bidirectional Mendelian Randomization (MR) approach to investigate the bidirectional causal relationships between WMH phenotypes (including WMH volume, FA, and MD) and cognitive abilities (such as cognitive performance, intelligence, and cognitive function).

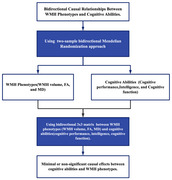

## 1 Introduction

White matter hyperintensities (WMHs), also referred to as white matter lesions or white matter changes, are abnormal high‐signal areas observed in the deep and subcortical white matter on magnetic resonance imaging (MRI). These areas appear bright white on T2‐weighted imaging or fluid‐attenuated inversion recovery (FLAIR) sequences, typically indicating myelin loss, axonal damage, or small vessel disease in the white matter, and representing macroscopic structural brain damage associated with various etiologies (Bauer et al. 2021). WMH is closely associated with hypertension, cerebrovascular diseases, and aging (Andere et al. 2022; Xie et al. 2024). Increasing evidence demonstrates the detrimental effects of WMH on the brain structure and function of older adults (Zeng et al. 2020; Lampe et al. 2019; Reed et al. 2004).

Cognitive abilities refer to the brain's capacity to process information, encompassing a range of complex psychological processes such as memory, attention, language, thinking, problem‐solving, and decision‐making. It is a fundamental component of daily life and social activities, with good cognitive abilities being essential for an individual's normal functioning (Frith et al. 2017). Given the aging population and the rising prevalence of neurodegenerative diseases, research on cognitive abilities has gained increasing importance (Gouela et al. 2024).

Existing research has demonstrated the close association between WMH volume and cognitive abilities. For instance, Du et al. (2024)’s study found that WMH partially mediated the relationship between frailty and cognitive impairment in patients with meningitis (MMD). In groups with comorbid HIV and alcohol use disorder (AUD), WMH volume significantly increased and was associated with declines in attention, working memory, executive function, and motor skills (Pfefferbaum et al. 2024). Xiao et al. (2024)’s research utilizing multimodal MRI and DTI parameter analysis discovered that white matter changes mediated the causal relationship between ventricular enlargement and cognitive decline in patients with hydrocephalus. van Etten et al. (2024) found that regional WMH volume mediated the relationship between APOE *ε*4 gene status and cognition in young older adults, with significant differences observed across age groups. Zhang et al.’s study showed that WMH volume was independently associated with cognitive impairment, particularly in visuospatial cognition and delayed recall. Furthermore, the ratio of WMH volume to total white matter volume was considered a strong predictor of cognitive impairment (Zhang et al. 2023). Morales et al. (2024)’s research found that increased frontal and parietal WMH volumes were associated with poorer cognitive performance in Alzheimer's disease (AD) prevention trials. Wang et al. (2022) revealed that patients with high WMH scores had reduced brain functional network efficiency, suggesting that brain network changes might be one of the mechanisms leading to cognitive impairment. Liao et al. (2024)’s study discovered that deep medullary vein (DMV) scores were associated with cognitive impairment in patients with cerebral small vessel disease (CSVD), with this association mediated by WMH and brain parenchymal fraction (BPF).

Despite the extensive research on the association between WMH and cognitive abilities, their causal relationship remains unclear. In this study, single nucleotide polymorphisms (SNPs) were carefully selected from a genome‐wide association study (GWAS) of MRI markers of small vessel disease in the brain based on 42,310 participants (Persyn et al. 2020), another GWAS involving 14 independent epidemiological cohorts of European ancestry and 9,295,118 genetic variants that passed quality control (Savage et al. 2018), and a large‐scale genetic association analysis of educational attainment in approximately 1.1 million samples (Lee et al. 2018). We aim to explore the causal relationship between WMH phenotypes and cognitive abilities using a bidirectional two‐sample Mendelian randomization (MR) approach, providing a new perspective on the interactive effects between the two.

Two‐sample bidirectional MR analysis uses genetic variants as instrumental variables, effectively avoiding the influence of confounding factors and thereby providing more reliable causal inferences (Davey Smith and Hemani 2014; Emdin, Khera, and Kathiresan 2017). In recent years, MR studies have garnered significant attention in the field of cognitive abilities. These studies have investigated the causal relationships between cognitive abilities and various factors such as midlife hypertension (Sun et al. 2020), vitamin D levels (Maddock et al. 2017), oxidative stress (Fan et al. 2024), kidney function (Richard et al. 2021), obesity (Norris et al. 2023), tobacco and cannabis use (Mahedy et al. 2021), educational attainment (Wang et al. 2021), blood glucose levels (Garfield et al. 2021), and coffee consumption (Zhou et al. 2018). By using genetic variants as instrumental variables, these studies provide more reliable evidence, revealing the potential impact of these factors on cognitive abilities. However, the interaction between cognitive abilities and white matter hyperintensity (WMH) phenotypes remains a novel area of investigation, necessitating further research to elucidate its complex relationship.

## 2 Methods

### 2.1 Study Design

The groundbreaking research by Davey Smith and Hemani (2014) laid the foundational framework for implementing two‐sample MR, allowing for the effective use of publicly available genetic summary data. Our study strictly adhered to the “Strengthening the Reporting of Observational Studies in Epidemiology‐Mendelian Randomization (STROBE‐MR)” guidelines published in the BMJ, formulated within the framework of the Enhancing the Quality and Transparency of Health Research (EQUATOR) network (Skrivankova et al. 2021). By integrating the STROBE‐MR guidelines into our methodology, we aim to enhance the clarity and rigor of our research assessment.

**FIGURE 1:**
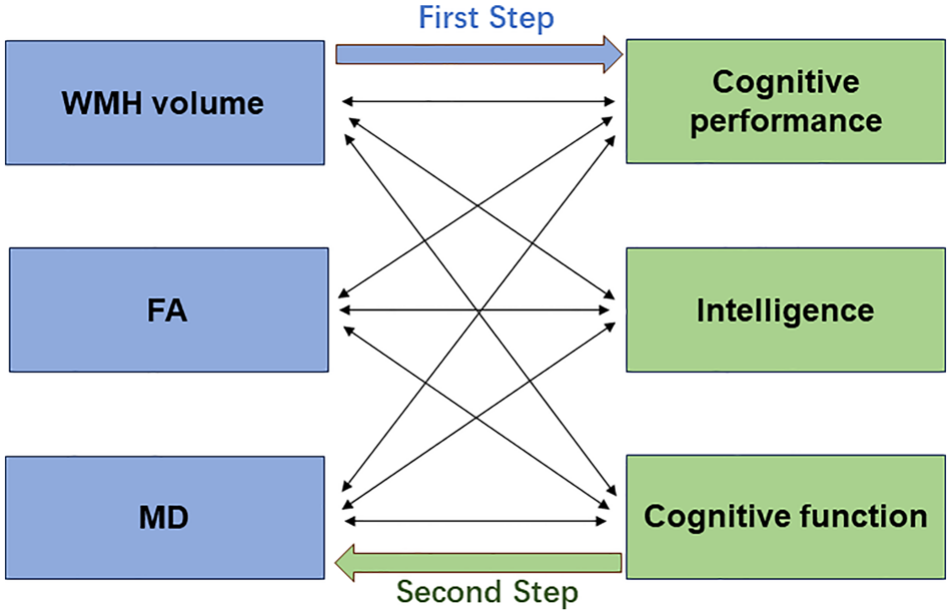
Bidirectional two‐sample Mendelian randomization study. FA, fractional anisotropy; MD, mean diffusivity.

This study employs a bidirectional two‐sample MR method, conducted in two main steps (illustrated in Figure 1):

#### 2.1.1 Step 1: Forward Causal Relationship Analysis

This step evaluates the relationship between WMH phenotypes, as determined by GWAS, as the exposure factor and cognitive abilities, as determined by GWAS, as the outcome.

#### 2.1.2 Step 2: Reverse Causal Relationship Analysis

To assess the possibility of reverse causality, a reverse MR analysis is conducted, using cognitive abilities as the exposure factor and WMH phenotypes as the outcome variable.

**FIGURE 2:**
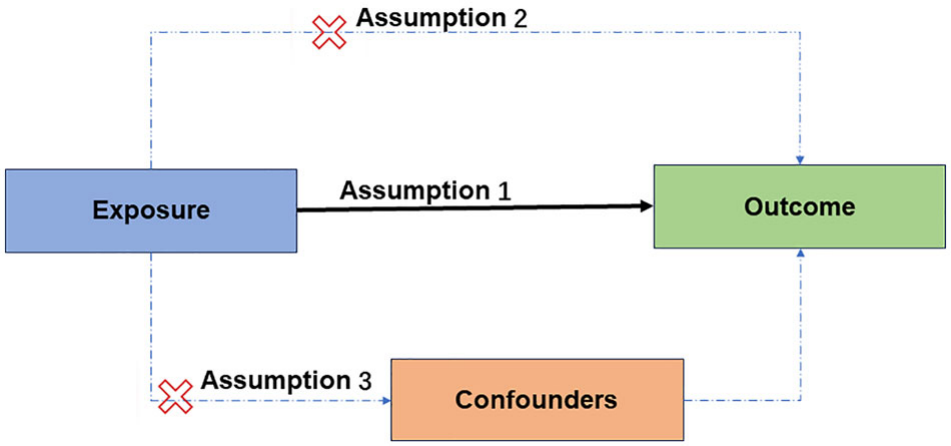
Core assumptions for selecting genetic variants.

To ensure the reliability of the analysis, this study adheres to the following three core assumptions in selecting genetic variants (illustrated in Figure 2):

1. Genetic variants should be strongly associated with the exposure factor.
2. Genetic variants should be associated with the outcome only through the exposure factor.
3. Genetic variants should be independent of potential confounders.

To meet the first assumption, significant and uncorrelated genetic variants were selected at a significance level of *p *< 5 × 10^−8^. For the evaluation of the latter two assumptions, sensitivity analyses were performed.

### 2.2 Data Sources

This study utilized publicly available summary statistics from GWAS, encompassing log‐transformed and normalized data on WMH volume from the UK Biobank. It also included the first principal component analysis (PCA) results of fractional anisotropy (FA) and mean diffusivity (MD) measurements across 48 brain regions. The three neuroimaging characteristics evaluated in this study—WMH volume, MD, and FA—were obtained from a comprehensive GWAS meta‐analysis encompassing 42,310 European individuals aged 40–69 years, derived from the UK Biobank, the CHARGE consortium, and the WMH‐Stroke study (PMID: 32358547) (Persyn et al. 2020). These datasets were produced following consistent neuroimaging protocols, utilizing Siemens Skyra 3.0T MRI scanners. To ensure the reliability of genetic analyses, WMH volume was subjected to normalization and log‐transformation, correcting for variations in individual brain volume. Specifically, WMH volume was standardized to total brain volume and logarithmically transformed to approximate a normal distribution. Stringent quality control measures were implemented to exclude individuals with central nervous system conditions linked to white matter anomalies, such as stroke, dementia, or multiple sclerosis. Analyses of MD and FA were confined to 17,467 and 17,663 participants from the UK Biobank, respectively. Centralized processing of DTI parameters across 48 distinct white matter regions was performed by the UK Biobank. To generate global metrics for MD and FA within the entire white matter, PCA was applied to these regions, retaining only those with significant contributions to the first principal component. This primary component was subsequently used in genome‐wide association analyses to investigate correlations with genetic variants.

Additionally, GWAS data on cognitive abilities, including cognitive performance, intelligence, and cognitive function, were incorporated into this study. Cognitive performance refers to scores obtained from standardized cognitive tasks, which are highly specific (PMID: 30038396) (Lee et al. 2018). The genetic data for cognitive performance were derived from the ebi‐a‐GCST006572 dataset (2018), encompassing 257,841 individuals of European ancestry (54% female, aged ≥30 years). Intelligence is defined as the capacity for reasoning, understanding, and learning (PMID: 29942086) (Savage et al. 2018). Corresponding data were sourced from the ebi‐a‐GCST006250 dataset (2018), which included 269,867 individuals of European ancestry (balanced gender, aged 6–100 years). Cognitive function, representing a composite measure of overall ability—including memory, attention, reasoning, and processing speed—was based on data from the ieu‐b‐4838 dataset (2022) (PMID: 35534559) (Howe et al. 2022). This dataset included 22,593 individuals of European ancestry, with details on age range and gender distribution not reported. Detailed sample sizes and the number of SNPs for each dataset are summarized in Table 1.

**TABLE 1:** Details of data sources included in the study.

Phenotypes	Sample size	Number of SNPs	Population	GWAS‐ID	PMID
WMH volume	42,310	18,381	European	GWAS_UKB_logWMHnorm	32358547
FA	42,310	17,663	European	GWAS_UKB_PC1_FA	32358547
MD	42,310	17,467	European	GWAS_UKB_PC1_MD	32358547
Cognitive performance	257,841	10,066,414	European	ebi‐a‐GCST006572	30038396
Intelligence	269,867	9,276,181	European	ebi‐a‐GCST006250	29942086
Cognitive function	22,593	6,719,661	European	ieu‐b‐4838	35534559

Abbreviations: FA, fractional anisotropy; MD, mean diffusivity; WMH, white matter hyperintensities.

### 2.3 Selection of Genetic Instrumental Variables

To further enhance the independence of the variables, we applied linkage disequilibrium criteria (*r* ^2^ = 0.001; 10 Mb). Additionally, we set a threshold to exclude SNPs with effect allele frequencies exceeding 0.420 to ensure that the selected SNPs could represent the majority of the population. To ensure the robustness of the IV selection process, we excluded SNPs with palindromic sequences and ambiguous intermediate effect frequencies. We also excluded SNPs with an *F*‐statistic less than 10 to assess the strength of the IVs.

### 2.4 Sensitivity Analysis

To detect the robustness of the analysis results, various sensitivity analyses were conducted, including

1. Using a more lenient inclusion threshold for SNPs of *p *< 1 × 10^−5^.
2. Examining the potential impact of pleiotropy (i.e., genetic variants affecting multiple traits) of the genetic instruments.
3. Using different methods to evaluate the validity of the genetic instruments and the robustness of the results.

Additionally, to further validate the robustness of the causal estimates, a leave‐one‐out analysis was conducted, whereby one SNP was excluded at a time to evaluate the stability of the MR results. Heterogeneity analysis was carried out to elucidate differences in causal effects across various subpopulations. The inverse variance weighted (IVW) method combined with Cochran's *Q* statistic was employed to assess heterogeneity, and the MR Egger intercept was utilized to evaluate pleiotropy among SNPs. Funnel plots and scatter plots were used to identify potential biases.

### 2.5 Statistical Analysis

The analysis was conducted using the two‐sample MR package (version 0.6.4) within the R programming environment (version 4.4.0). We utilized multiple two‐sample MR methods, including MR Egger, weighted median, IVW, simple mode, and weighted mode. Exposure factors were standardized by calculating standard deviation increments, and odds ratios (OR) were used to assess the association between exposure and outcome. Sensitivity, heterogeneity, threshold change, and bidirectional analyses were performed to evaluate the causal relationship between exposure factors and outcome risks.

## 3 Results

### 3.1 Step 1: Forward Causal Relationship Analysis

#### 3.1.1 Effect of WMH Phenotypes on Cognitive Abilities

**FIGURE 3:**
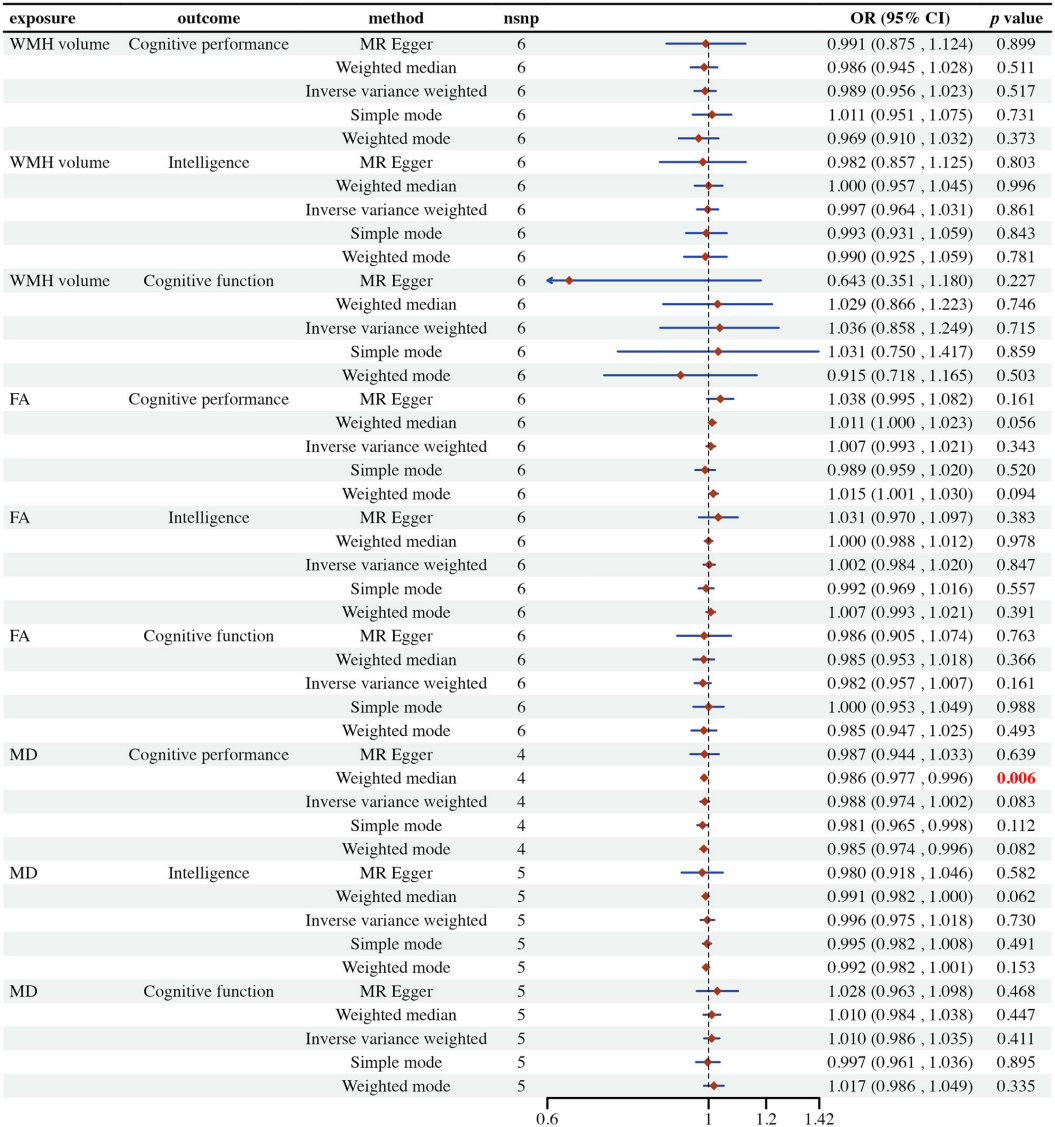
Mendelian randomization estimates of the association between white matter hyperintensity volume and cognitive function. Figures S1–S12: scatter plot (A), forest plot (B), “leave‐one‐out” analysis (C), and funnel plot (D), for MR analysis. FA, fractional anisotropy; MD, mean diffusivity; WMH, white matter hyperintensities.

This study employed MR analysis to explore the potential causal relationships between WMH volume, FA, and MD with cognitive performance, intelligence, and cognitive function. The findings indicate that these relationships are not statistically significant (illustrated in Figure 3). Specifically, most MR methods used, including MR Egger, weighted median, IVW, simple mode, and weighted mode, yielded *p* values greater than 0.05, indicating nonsignificance and wide confidence intervals, which suggest substantial heterogeneity and uncertainty in the data. A few significant findings, such as the relationship between MD and cognitive performance using the weighted median method, which showed a significant negative correlation with a *p* value of 0.006, were not consistently supported across different methods. These results imply that the potential causal effects of WMH, FA, and MD on cognitive outcomes may be negligible or undetectable due to data heterogeneity and insufficient statistical power.

#### 3.1.2 Study Evaluation of Heterogeneity and Pleiotropy

The heterogeneity test results for WMH volume with cognitive performance and intelligence show no significant heterogeneity, and horizontal pleiotropy is also not significant, indicating consistent associations across different MR methods (illustrated in Table 2). However, the relationship between WMH volume and cognitive function shows significant heterogeneity in the IVW method (*Q* = 14.807, *p *= 0.011). For FA, the heterogeneity test results for cognitive performance (*Q* = 9.542, *p *= 0.049) and intelligence (*Q* = 21.769, *p* < 0.001) are significant, but horizontal pleiotropy is not, suggesting that pleiotropy is not a major issue. The heterogeneity and horizontal pleiotropy tests for FA and cognitive function are not significant. MD shows significant heterogeneity in its association with cognitive performance (*Q* = 8.606, *p *= 0.014) and intelligence (*Q* = 31.056, *p* < 0.001), but horizontal pleiotropy is not significant, and the results for cognitive function are consistent. These findings indicate that although there is data heterogeneity in the relationships between WMH, FA, and MD with cognitive outcomes, horizontal pleiotropy is generally not significant. Further research should consider the issue of data heterogeneity.

**TABLE 2:** Heterogeneity test and horizontal pleiotropy test results of the association between white matter hyperintensity volume and cognitive function.

Exposure		MR heterogeneity test				MR horizontal pleiotropy test		
	Outcome	Method	*Q*	*Q*‐df	*Q*‐pval	Egger intercept	SE	*p* value
WMH volume	Cognitive performance	MR Egger	2.156	4.000	0.707	0.000	0.005	0.968
		Inverse variance weighted	2.158	5.000	0.827			
WMH volume	Intelligence	MR Egger	4.866	4.000	0.301	0.001	0.005	0.828
		Inverse variance weighted	4.931	5.000	0.424			
WMH volume	Cognitive function	MR Egger	9.037	4.000	0.060	0.039	0.024	0.185
		Inverse variance weighted	14.807	5.000	0.011			
FA	Cognitive performance	MR Egger	9.542	4.000	0.049	−0.011	0.007	0.214
		Inverse variance weighted	14.741	5.000	0.012			
FA	Intelligence	MR Egger	21.769	4.000	0.000	−0.010	0.010	0.389
		Inverse variance weighted	26.847	5.000	0.000			
FA	Cognitive function	MR Egger	2.162	4.000	0.706	−0.001	0.015	0.923
		Inverse variance weighted	2.173	5.000	0.825			
MD	Cognitive performance	MR Egger	8.606	2.000	0.014	0.000	0.010	0.984
		Inverse variance weighted	8.608	3.000	0.035			
MD	Intelligence	MR Egger	31.056	3.000	0.000	0.007	0.013	0.627
		Inverse variance weighted	34.062	4.000	0.000			
MD	Cognitive function	MR Egger	0.279	3.000	0.964	−0.008	0.013	0.611
		Inverse variance weighted	0.599	4.000	0.963			

Abbreviations: Q, quantile; df, degrees of freedom; SE, standard error; WMH, white matter hyperintensities; FA, fractional anisotropy; MD, mean diffusivity.

### 3.2 Step 2: Reverse Causal Relationship Analysis

#### 3.2.1 Effect of Cognitive Abilities on WMH Phenotypes

The analysis revealed that cognitive performance and WMH volume had no significant causal relationships across all methods, with *p* values exceeding 0.05 (illustrated in Table 3). Similarly, the association between cognitive performance and FA was not significant across all analytical methods, with *p* values also greater than 0.05. In the case of cognitive performance and MD, most methods did not indicate significant causal relationships, although the IVW method showed a *p* value approaching significance (*p *= 0.068). Overall, these findings suggest that the associations between cognitive performance and WMH, FA, and MD are not statistically significant, indicating that the potential causal effects of these metrics on cognitive performance are likely minimal or nonsignificant.

**TABLE 3:** Mendelian randomization estimates of the association between cognitive function and white matter hyperintensity volume.

Exposure	Outcome	Method	*n*SNP	*p* value	OR (95%CI)
Cognitive performance	WMH volume				
		MR Egger	127	0.966124	1.009 (0.674–1.509)
		Weighted median	127	0.473726	0.955 (0.843–1.083)
		Inverse variance weighted	127	0.324199	0.956 (0.874–1.045)
		Simple mode	127	0.942972	0.988 (0.713–1.369)
		Weighted mode	127	0.689339	0.936 (0.676–1.295)
Cognitive performance	FA				
		MR Egger	117	0.593406	0.604 (0.095–3.826)
		Weighted median	117	0.259939	1.392 (0.783–2.475)
		Inverse variance weighted	117	0.523373	1.14 (0.762–1.706)
		Simple mode	117	0.184004	3.303 (0.573–19.046)
		Weighted mode	117	0.243849	2.958 (0.482–18.165)
Cognitive performance	MD				
		MR Egger	122	0.973781	0.97 (0.162–5.799)
		Weighted median	122	0.052697	0.574 (0.327–1.006)
		Inverse variance weighted	122	0.068047	0.685 (0.457–1.028)
		Simple mode	122	0.150415	0.31 (0.064–1.514)
		Weighted mode	122	0.167615	0.333 (0.07–1.573)

Abbreviations: CI, confidence interval; FA, fractional anisotropy; MD, mean diffusivity; OR, odds ratio; SNP, single‐nucleotide polymorphisms; WMH, white matter hyperintensities.

Unfortunately, we were unable to conduct this part of the analysis as we did not find suitable instrumental variables for the reverse analysis, such as intelligence and cognitive function.

#### 3.2.2 Study Evaluation of Heterogeneity and Pleiotropy

The findings presented in Table 4 reveal an absence of significant heterogeneity or horizontal pleiotropy in the associations between cognitive performance and WMH volume, FA, and MD. These results imply that the MR methods employed in these analyses demonstrate a high level of consistency and reliability.

**TABLE 4:** Heterogeneity test and horizontal pleiotropy test results of the association between cognitive function and white matter hyperintensity volume.

Exposure		MR heterogeneity test				MR horizontal pleiotropy test		
	Outcome	Method	*Q*	Q‐df	Q‐pval	Egger intercept	SE	*p* value
Cognitive performance	WMH volume	MR Egger	76.300	125.000	1.000	−0.001	0.004	0.789
		Inverse variance weighted	76.371	126.000	1.000			
Cognitive performance	FA	MR Egger	86.162	115.000	0.980	0.014	0.020	0.491
		Inverse variance weighted	86.640	116.000	0.981			
Cognitive performance	MD	MR Egger	78.451	120.000	0.999	−0.008	0.019	0.696
		Inverse variance weighted	78.604	121.000	0.999			

Abbreviations: df, degrees of freedom; FA, fractional anisotropy; MD, mean diffusivity; Q, Quantile; SE, standard error; WMH, white matter hyperintensities.

## 4 Discussion

In this study, we employed a two‐sample MR analysis utilizing data from GWAS to explore the potential causal pathway linking WMH phenotypes (WMH volume, FA, MD) and cognitive abilities (cognitive performance, intelligence, and cognitive function). Our findings suggest the absence of a significant causal association between WMH volume, FA, MD, and cognitive abilities. This assertion is corroborated by the nonsignificant *p* values and broad confidence intervals derived from the majority of MR methods employed in our investigation. Furthermore, our assessments for heterogeneity did not uncover notable heterogeneity or pleiotropy concerning WMH volume and cognitive performance or intelligence. Nonetheless, we did observe significant heterogeneity in the associations between WMH volume and cognitive function, FA and cognitive performance and intelligence, as well as MD and cognitive performance and intelligence. Similarly, reverse analyses did not reveal substantial causal relationships, which further bolsters the argument against the presence of a bidirectional causal relationship between WMH phenotypes and cognitive abilities.

Previous investigations have suggested a potential link between the volume of WMH and cognitive abilities (Xu et al. 2023; Hannawi et al. 2024; Seiler et al. 2018; Zwartbol et al. 2022; Zhang et al. 2023). However, the current study was unable to provide definitive evidence to establish a clear causal relationship between these variables. It remains plausible that WMH volume, FA, and MD contribute to cognitive decline. Rather than serving as direct causative factors, these parameters may function as indicative biomarkers of cognitive deterioration. These observations underscore the complexity of biological mechanisms and the interplay of various influencing factors. Cognitive abilities and WMH alterations may arise from common risk factors, such as vascular abnormalities (e.g., hypertension and arteriosclerosis), metabolic disorders (e.g., diabetes and obesity), inflammatory activity, and endothelial dysfunction (Parfenov et al. 2019; Botz, Lohner, and Schirmer 2023). These shared risk elements are likely to impact brain structure and function through multiple pathological mechanisms. Additionally, the frequent coexistence of WMH and cognitive impairment may imply their combined contribution to wider neuropathological processes. WMH is commonly found alongside other brain abnormalities, including cerebrovascular damage, brain atrophy, and ventricular dilation, which collectively worsen cognitive decline (Silbert et al. 2008; Kloppenborg et al. 2012). Moreover, cognitive reserve and psychological resilience—though not directly addressed in this study—are likely critical in mitigating the negative effects of cerebrovascular damage and preserving cognitive capabilities (Arola et al. 2021).

Previous research has highlighted a notable correlation between the complexity of WMH shape, distribution, and cognitive decline, highlighting their potential as neuroimaging markers for brain function impairment (Zwartbol et al. 2022). A subsequent study involving 3077 elderly individuals revealed that irregular WMH shapes and larger volumes were linked to an elevated risk of long‐term dementia (Keller et al. 2023). The initial frontal and corpus callosum knee WMH volumes were autonomous predictors of long‐term cognitive impairment (Li et al. 2023). Asymmetry in WMH volume in the parietal and temporal lobes was associated with functional decline, and especially higher WMH volume asymmetry in the left parietal lobe was independently linked to accelerated functional decline (Dhamoon et al. 2017). The results emphasize the significant influence of WMH shape and distribution on the assessment of cognitive decline. Additionally, growing evidence indicates that the use of a single parameter, such as WMH volume, does not suffice to fully explain causal relationships. A thorough understanding of changes in cognitive abilities requires analyses that incorporate multidimensional and complex attributes.

The wide confidence intervals and variations in *p* values among different methods in this study underscore the complexity and heterogeneity of the data. This suggests that the relationship between these neuroimaging markers and cognitive outcomes may be influenced by other confounding factors, or that the true effects are weak and require larger, more robust datasets for detection. The significant heterogeneity in the associations of WMH, FA, and MD with certain cognitive outcomes indicates that the heterogeneity of the data may impact the reliability of the findings. This heterogeneity could be due to population stratification, differences in measurement techniques, and unmeasured confounders. Although most analyses did not reveal significant levels of horizontal pleiotropy, indicating that pleiotropic effects are not substantial, the observed heterogeneity should be interpreted cautiously. The significant heterogeneity observed in the relationships between WMH volume and cognitive function, as well as FA/MD and cognitive performance and intelligence, highlights the need for further research.

This study's strength lies in its contribution to the literature by advancing our understanding of the causal relationship between WMH, FA, MD, and cognitive abilities, as previous research has predominantly focused on the correlation between these factors. Additionally, this study is the first to utilize MR methods, employing genetic variation as instrumental variables to deduce the causal relationship between WMH phenotypes and cognitive abilities. The study's methodology is comprehensive, as it utilizes a two‐sample MR method, following the transparency and reproducibility guidelines for MR reporting. This methodology effectively mitigates the impact of confounding factors and reverse causation.

This study has several limitations. First, there is significant heterogeneity in the data regarding the associations between WMH phenotypes and cognitive function, as well as FA and cognitive performance and intelligence. This heterogeneity may affect the reliability of the results and requires cautious interpretation. Second, most MR methods yielded nonsignificant *p* values and wide confidence intervals, indicating that the causal relationship between WMH phenotypes and cognitive abilities may be small or nonexistent, limiting the conclusions of the study. Additionally, despite controlling for multiple confounding variables, unmeasured factors such as genetic predisposition and lifestyle factors may still influence the relationship between WMH volume and cognitive abilities, warranting further investigation. Moreover, the sample diversity is limited, especially regarding ethnic composition, as this study primarily focuses on European populations. Future research should aim for larger and more diverse samples to enhance the generalizability of the results. Lastly, this study only analyzed a single factor, and future studies should consider multifactor joint analysis to comprehensively understand the complex interaction between neuroimaging markers and cognitive outcomes.

Future research should emphasize increasing sample sizes and employing more sophisticated analytical techniques to enhance the understanding of the potential causal relationship between neuroimaging markers and cognitive outcomes. Furthermore, considering additional potential confounding variables and utilizing more comprehensive datasets can assist in clarifying these relationships and drawing more definitive conclusions. Although this study does not provide direct evidence of a potential causal relationship between neuroimaging biomarkers and cognitive outcomes, further research is required to explore this association. Integrating the Resilience and Reserve Framework into upcoming research provides essential perspectives on how variations in brain reserve and resilience influence cognitive abilities in the context of neuropathological changes. By addressing the limitations of current research and leveraging its strengths, future studies can achieve a more comprehensive understanding of the intricate interaction between brain structure and cognitive abilities.

## 5 Conclusion

Although this study did not identify significant causal relationships, it does not dismiss the potential impact of WMH volume, FA, and MD, on cognitive decline. Subsequent research should incorporate multifactorial joint analysis to gain a more comprehensive understanding of the intricate interplay between neuroimaging markers and cognitive outcomes.

## Author Contributions

**Xiaolu Ren**: conceptualization, methodology, investigation, data curation, writing–original draft, writing–review and editing. **Ting Li**: supervision, formal analysis, project administration, visualization. **Fang Li**: software, data curation, methodology, investigation. **Shan Liu**: conceptualization, methodology, resources, project administration.

## Ethics Statement

Ethical review and approval were not necessary for this study involving human participants, as it adhered to local legislation and institutional requirements. Written informed consent for participation was also not required for this study in accordance with national legislation and institutional requirements.

## Consent

This study does not involve human participants.

## Conflicts of Interest

The authors declare no conflicts of interest.

### Peer Review

The peer review history for this article is available at https://publons.com/publon/10.1002/brb3.70313.

## Supporting information



Figure S1 Scatter plot (A), forest plot (B), and “leave‐one‐out” analysis (C) for MR analysis of WMH volume and cognitive performance, funnel plot (D).
Figure S2 Scatter plot (A), forest plot (B), and “leave‐one‐out” analysis (C) for MR analysis of WMH volume and intelligence, funnel plot (D).
Figure S3 Scatter plot (A), forest plot (B), and “leave‐one‐out” analysis (C) for MR analysis of WMH volume and cognitive function, funnel plot (D).
Figure S4 Scatter plot (A), forest plot (B), and “leave‐one‐out” analysis (C) for MR analysis of FA and cognitive performance, funnel plot (D).
Figure S5 Scatter plot (A), forest plot (B), and “leave‐one‐out” analysis (C) for MR analysis of FA and intelligence, funnel plot (D).
Figure S6 Scatter plot (A), forest plot (B), and “leave‐one‐out” analysis (C) for MR analysis of FA and cognitive function, funnel plot (D).
Figure S7 Scatter plot (A), forest plot (B), and “leave‐one‐out” analysis (C) for MR analysis of MD and cognitive performance, funnel plot (D).
Figure S8 Scatter plot (A), forest plot (B), and “leave‐one‐out” analysis (C) for MR analysis of MD and intelligence, funnel plot (D).
Figure S9 Scatter plot (A), forest plot (B), and “leave‐one‐out” analysis (C) for MR analysis of MD and cognitive function, funnel plot (D).
Figure S10 Scatter plot (A), forest plot (B), and “leave‐one‐out” analysis (C) for MR analysis of cognitive performance and WMH volume, funnel plot (D).
Figure S11 Scatter plot (A), forest plot (B), and “leave‐one‐out” analysis (C) for MR analysis of cognitive performance and FA, funnel plot (D).
Figure S12 Scatter plot (A), forest plot (B), and “leave‐one‐out” analysis (C) for MR analysis of cognitive performance and MD, funnel plot (D).

## Data Availability

The original contributions presented in the study are included in the article. Further inquiries can be directed to the corresponding authors.
