# Supplementary material for: The Interaction Between Cognitive Abilities and White Matter Hyperintensity Phenotypes: A Novel Perspective on Bidirectional Causality
Source: Brain Behav. 2025 Feb 9;15(2):e70313. doi: 10.1002/brb3.70313 (PMC11808190; doi:10.1002/brb3.70313)
Supplement: Supplementary file 1 — Figure S1 Scatter plot (A), forest plot (B), and “leave‐one‐out” analysis (C) for MR analysis of WMH volume and cognitive performance, funnel plot (D). Figure S2 Scatter plot (A), forest plot (B), and “leave‐one‐out” analysis (C) for MR analysis of WMH volume and intelligence, funnel plot (D). Figure S3 Scatter plot (A), forest plot (B), and “leave‐one‐out” analysis (C) for MR analysis of WMH volume and cognitive function, funnel plot (D). Figure S4 Scatter plot (A), forest plot (B), and “leave‐one‐out” analysis (C) for MR analysis of FA and cognitive performance, funnel plot (D). Figure S5 Scatter plot (A), forest plot (B), and “leave‐one‐out” analysis (C) for MR analysis of FA and intelligence, funnel plot (D). Figure S6 Scatter plot (A), forest plot (B), and “leave‐one‐out” analysis (C) for MR analysis of FA and cognitive function, funnel plot (D). Figure S7 Scatter plot (A), forest plot (B), and “leave‐one‐out” analysis (C) for MR analysis of MD and cognitive performance, funnel plot (D). Figure S8 Scatter plot (A), forest plot (B), and “leave‐one‐out” analysis (C) for MR analysis of MD and intelligence, funnel plot (D). Figure S9 Scatter plot (A), forest plot (B), and “leave‐one‐out” analysis (C) for MR analysis of MD and cognitive function, funnel plot (D). Figure S10 Scatter plot (A), forest plot (B), and “leave‐one‐out” analysis (C) for MR analysis of cognitive performance and WMH volume, funnel plot (D). Figure S11 Scatter plot (A), forest plot (B), and “leave‐one‐out” analysis (C) for MR analysis of cognitive performance and FA, funnel plot (D). Figure S12 Scatter plot (A), forest plot (B), and “leave‐one‐out” analysis (C) for MR analysis of cognitive performance and MD, funnel plot (D). [file BRB3-15-e70313-s001.docx]

The Interaction Between Cognitive Function and White Matter Hyperintensity Volume: A Novel Perspective on Bidirectional Causality

**Step 1: Forward Causal Relationship Analysis：**

**Supplementary Figure 1.** Scatter plot (A), forest plot (B), and “leave-one-out” analysis (C) for MR analysis of WMH Volume and Cognitive performance, funnel plot (D).

**Supplementary Figure 2.** Scatter plot (A), forest plot (B), and “leave-one-out” analysis (C) for MR analysis of WMH Volume and Intelligence, funnel plot (D).

**Supplementary Figure 3.** Scatter plot (A), forest plot (B), and “leave-one-out” analysis (C) for MR analysis of WMH Volume and Cognitive Function, funnel plot (D).

**Supplementary Figure 4.** Scatter plot (A), forest plot (B), and “leave-one-out” analysis (C) for MR analysis of FA and Cognitive performance, funnel plot (D).

**Supplementary Figure 5.** Scatter plot (A), forest plot (B), and “leave-one-out” analysis (C) for MR analysis of FA and Intelligence, funnel plot (D).

**Supplementary Figure 6.** Scatter plot (A), forest plot (B), and “leave-one-out” analysis (C) for MR analysis of FA and Cognitive Function, funnel plot (D).

**Supplementary Figure 7.** Scatter plot (A), forest plot (B), and “leave-one-out” analysis (C) for MR analysis of MD and Cognitive performance, funnel plot (D).

**Supplementary Figure 8.** Scatter plot (A), forest plot (B), and “leave-one-out” analysis (C) for MR analysis of MD and Intelligence, funnel plot (D).

**Supplementary Figure 9.** Scatter plot (A), forest plot (B), and “leave-one-out” analysis (C) for MR analysis of MD and Cognitive Function, funnel plot (D).

**Step 2: Reverse Causal Relationship Analysis：**

**Supplementary Figure 10.** Scatter plot (A), forest plot (B), and “leave-one-out” analysis (C) for MR analysis of Cognitive performance and WMH Volume, funnel plot (D).

**Supplementary Figure 11.** Scatter plot (A), forest plot (B), and “leave-one-out” analysis (C) for MR analysis of Cognitive performance and FA, funnel plot (D).

**Supplementary Figure 12.** Scatter plot (A), forest plot (B), and “leave-one-out” analysis (C) for MR analysis of Cognitive performance and MD, funnel plot (D).


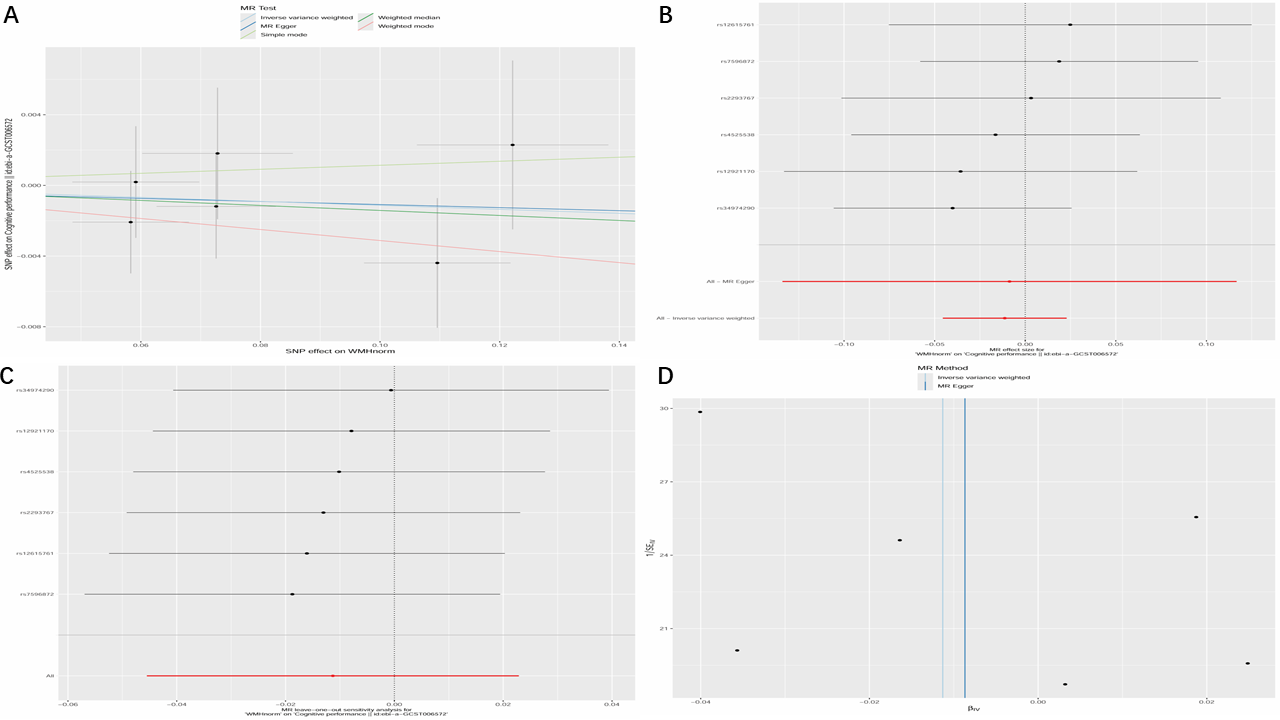


**Supplementary Figure 1.** Scatter plot (A), forest plot (B), and “leave-one-out” analysis (C) for MR analysis of WMH Volume and Cognitive performance, funnel plot (D).


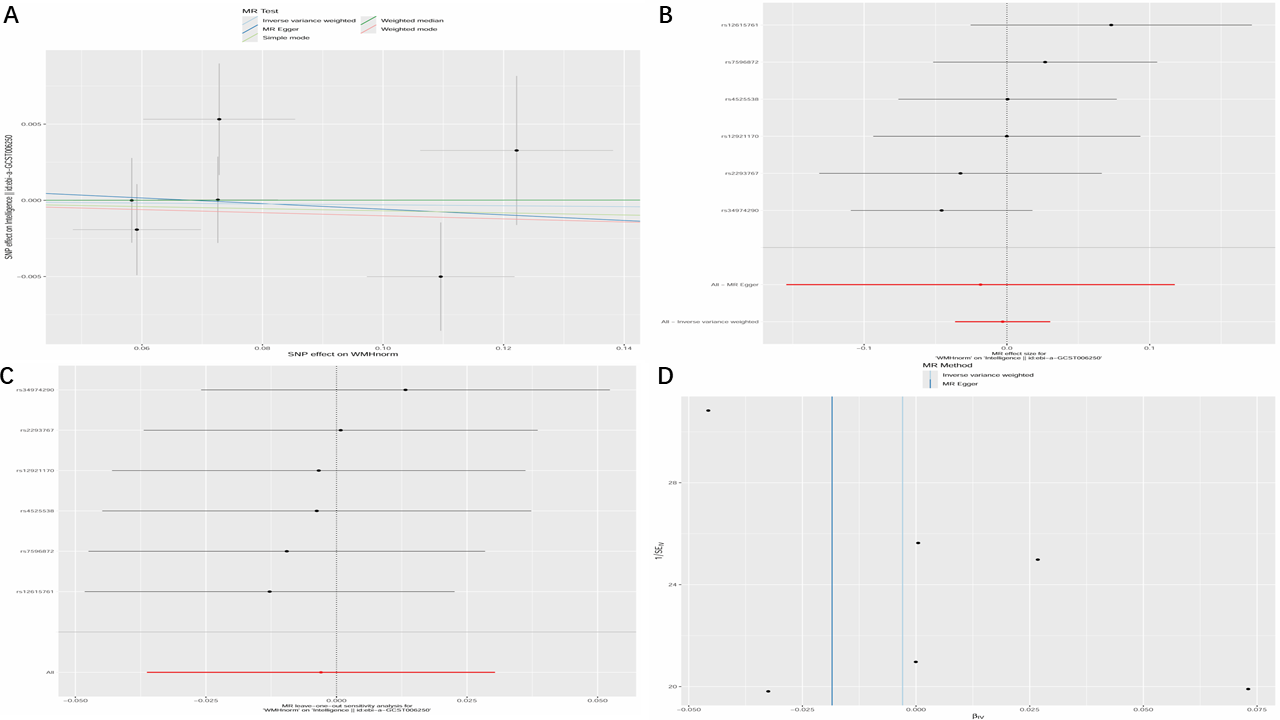


**Supplementary Figure 2.** Scatter plot (A), forest plot (B), and “leave-one-out” analysis (C) for MR analysis of WMH Volume and Intelligence, funnel plot (D).


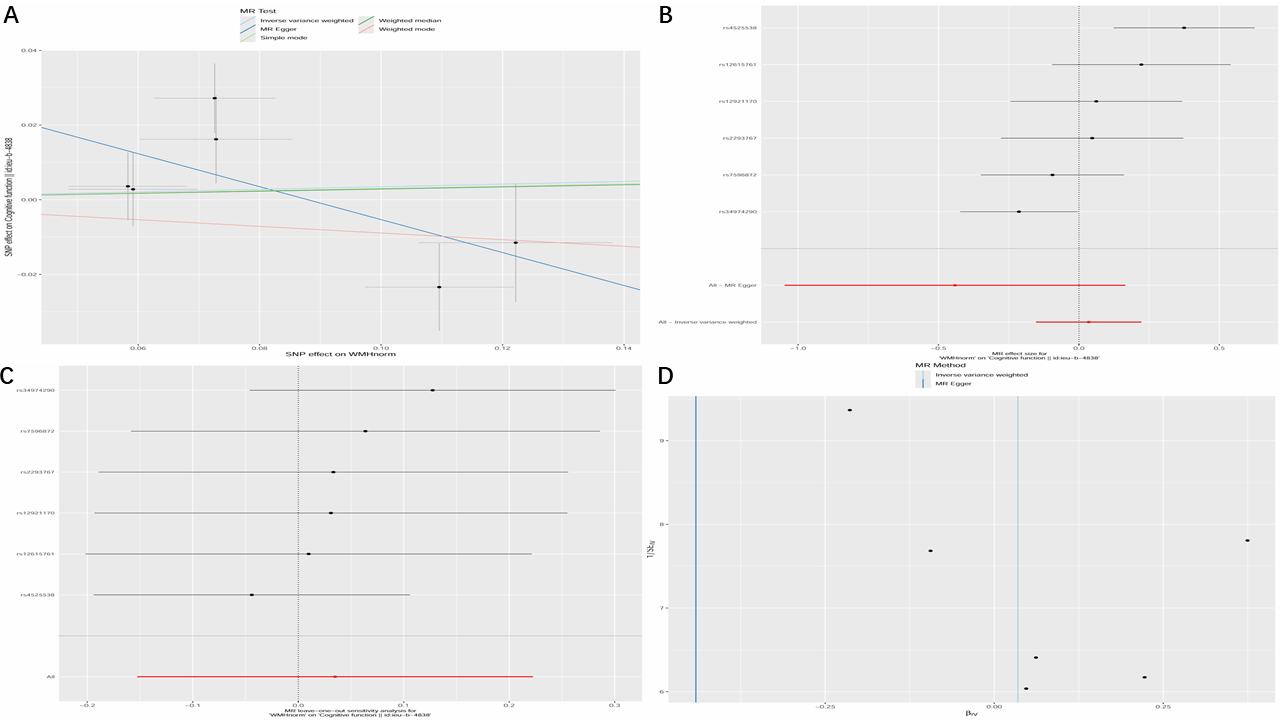


**Supplementary Figure 3.** Scatter plot (A), forest plot (B), and “leave-one-out” analysis (C) for MR analysis of WMH Volume and Cognitive Function, funnel plot (D).


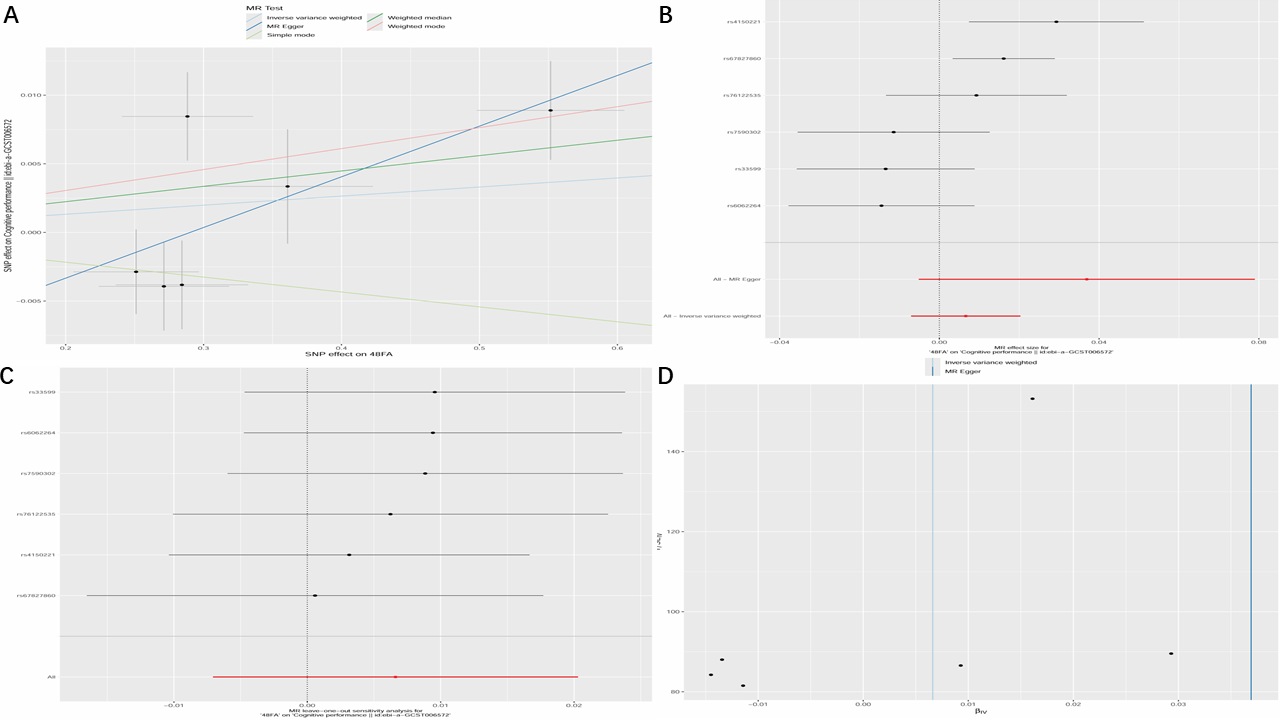


**Supplementary Figure 4.** Scatter plot (A), forest plot (B), and “leave-one-out” analysis (C) for MR analysis of FA and Cognitive performance, funnel plot (D).


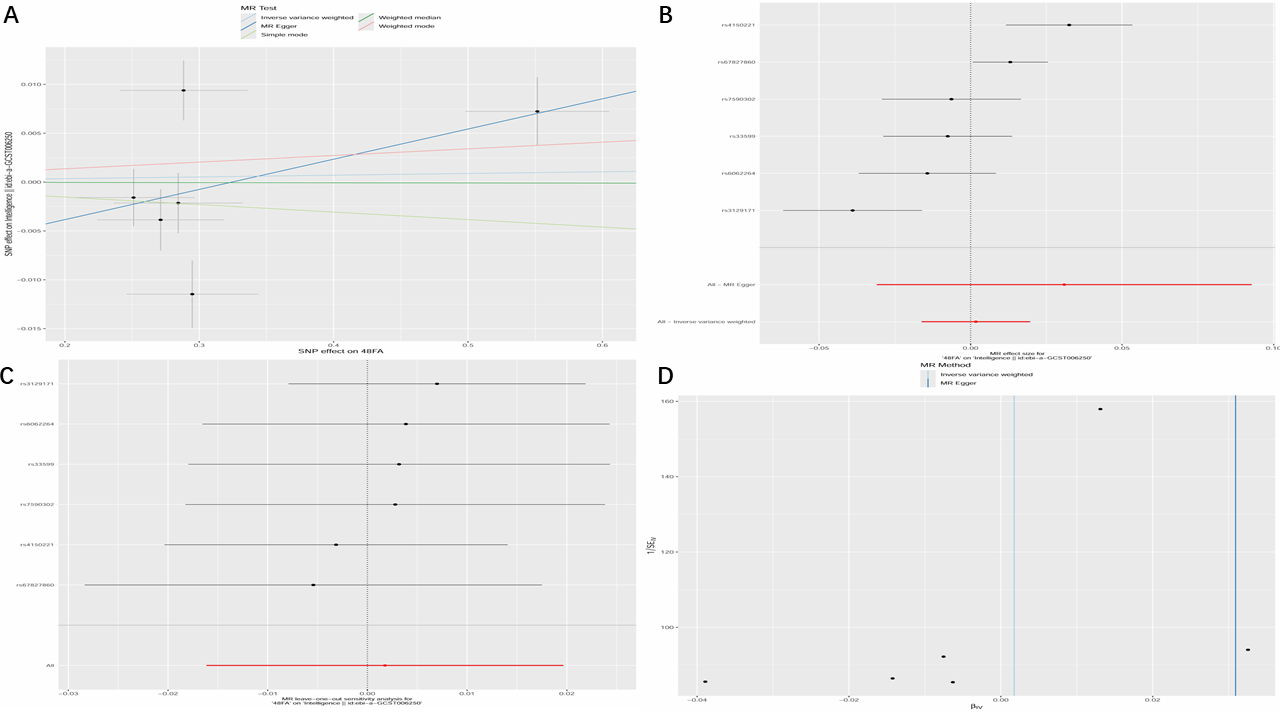


**Supplementary Figure 5.** Scatter plot (A), forest plot (B), and “leave-one-out” analysis (C) for MR analysis of FA and Intelligence, funnel plot (D).


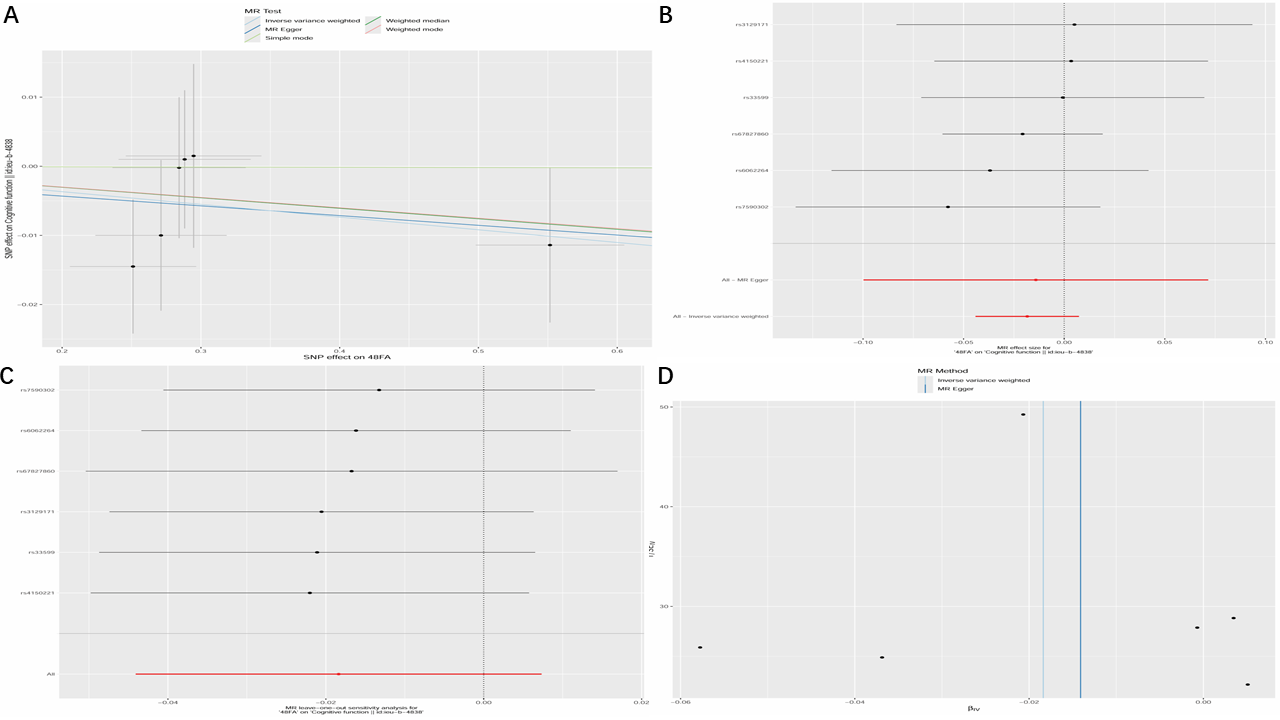


**Supplementary Figure 6.** Scatter plot (A), forest plot (B), and “leave-one-out” analysis (C) for MR analysis of FA and Cognitive Function, funnel plot (D).


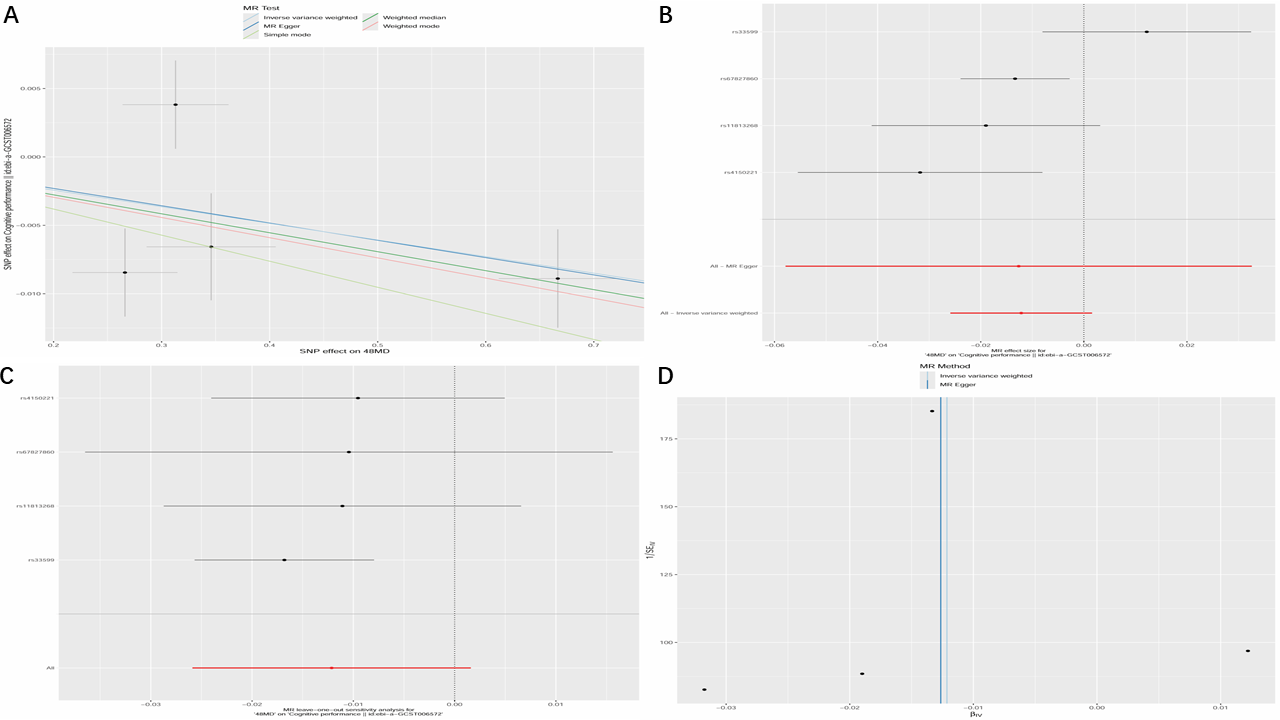


**Supplementary Figure 7.** Scatter plot (A), forest plot (B), and “leave-one-out” analysis (C) for MR analysis of MD and Cognitive performance, funnel plot (D).


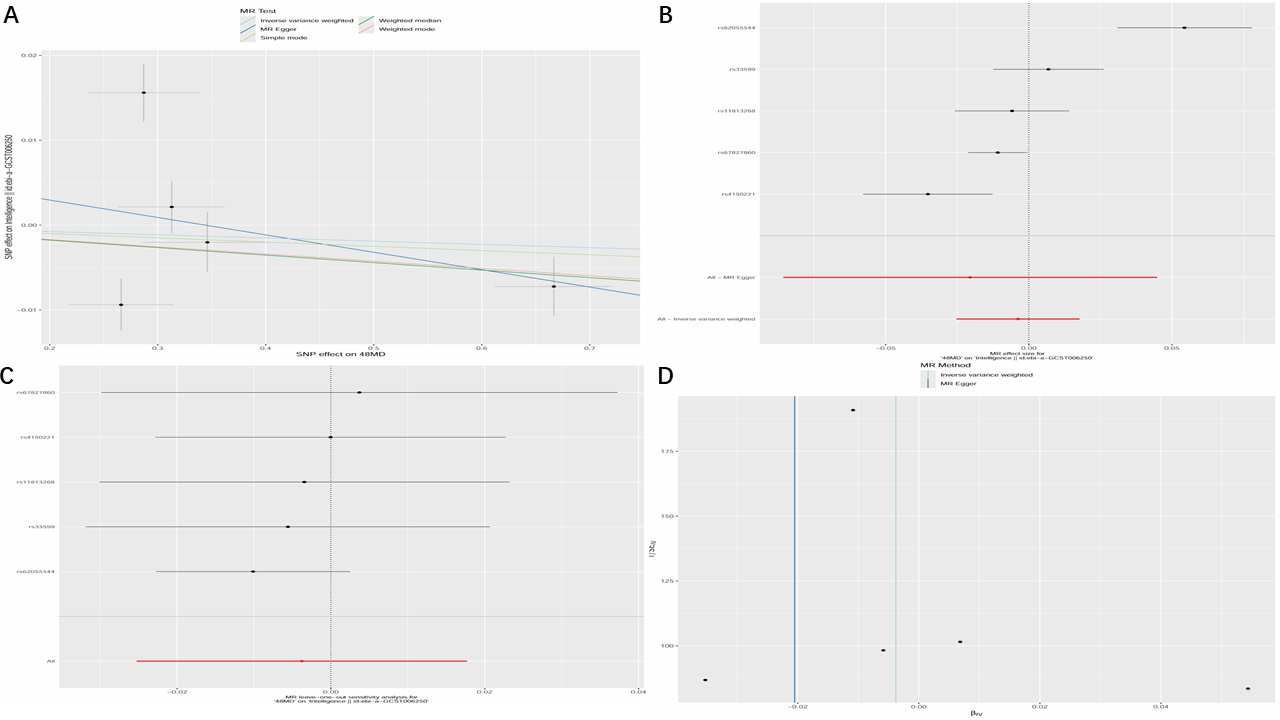


**Supplementary Figure 8.** Scatter plot (A), forest plot (B), and “leave-one-out” analysis (C) for MR analysis of MD and Intelligence, funnel plot (D).


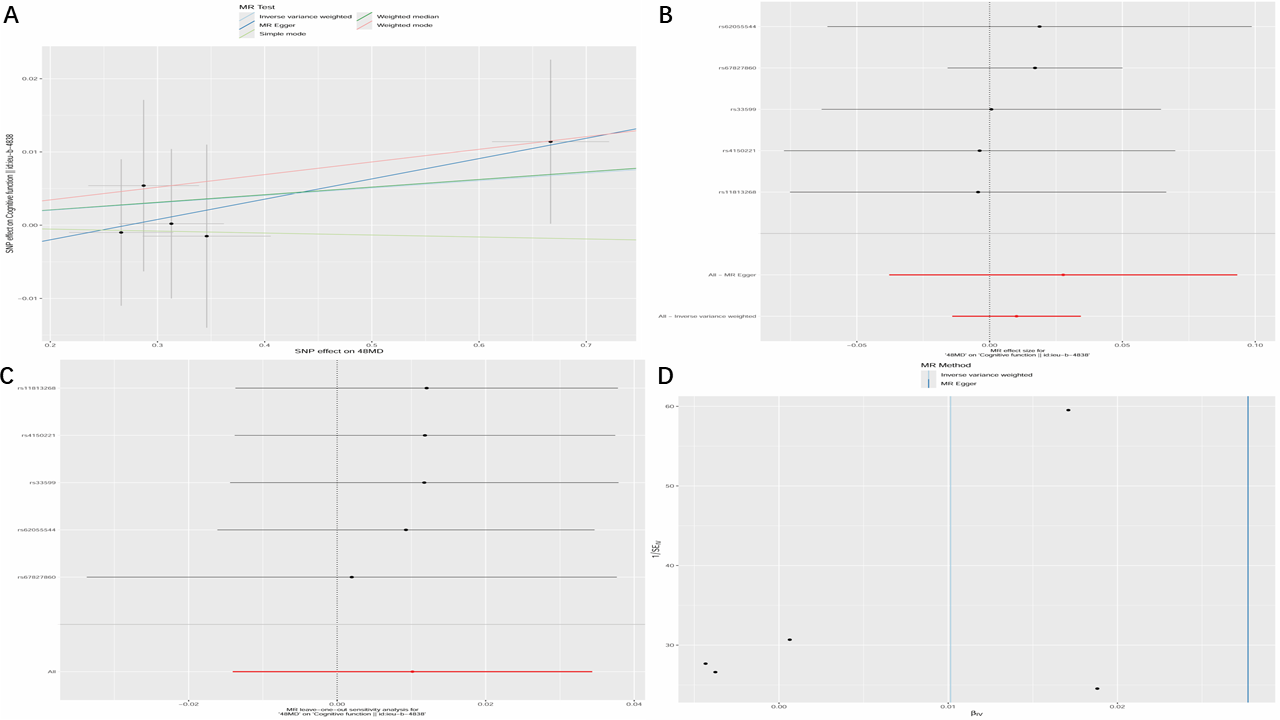


**Supplementary Figure 9.** Scatter plot (A), forest plot (B), and “leave-one-out” analysis (C) for MR analysis of MD and Cognitive Function, funnel plot (D).


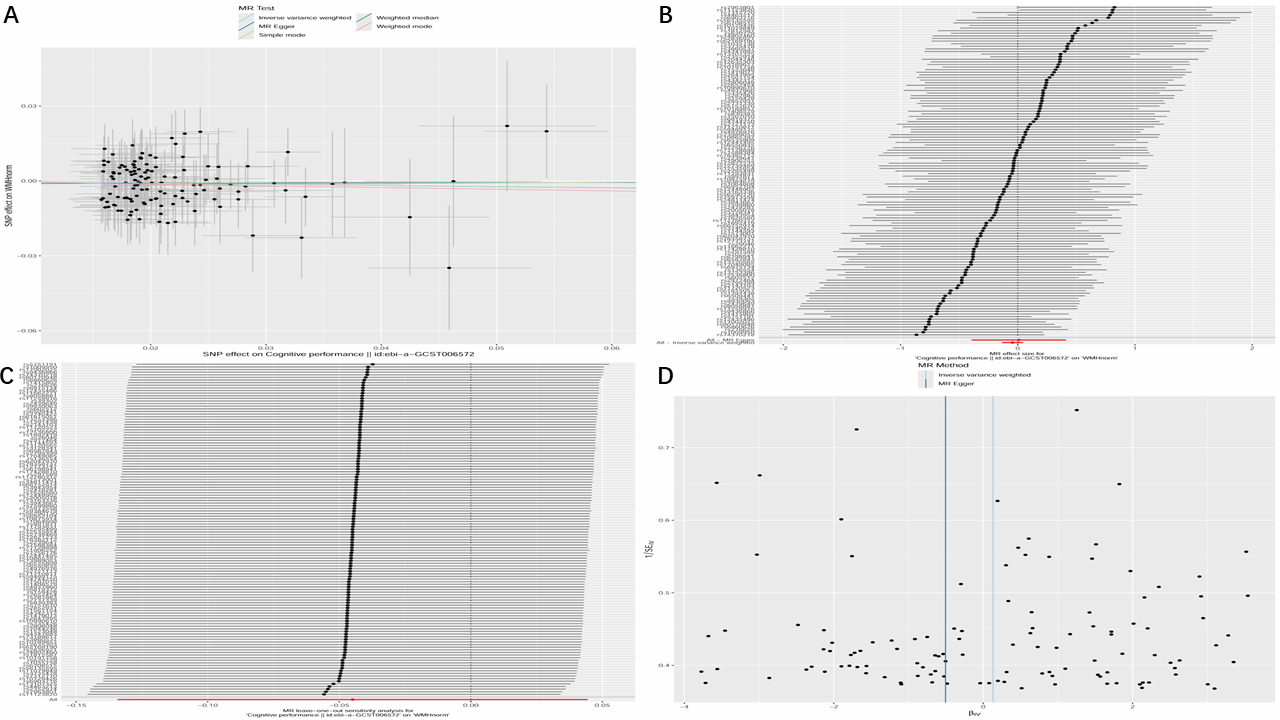


**Supplementary Figure 10.** Scatter plot (A), forest plot (B), and “leave-one-out” analysis (C) for MR analysis of Cognitive performance and WMH Volume, funnel plot (D).


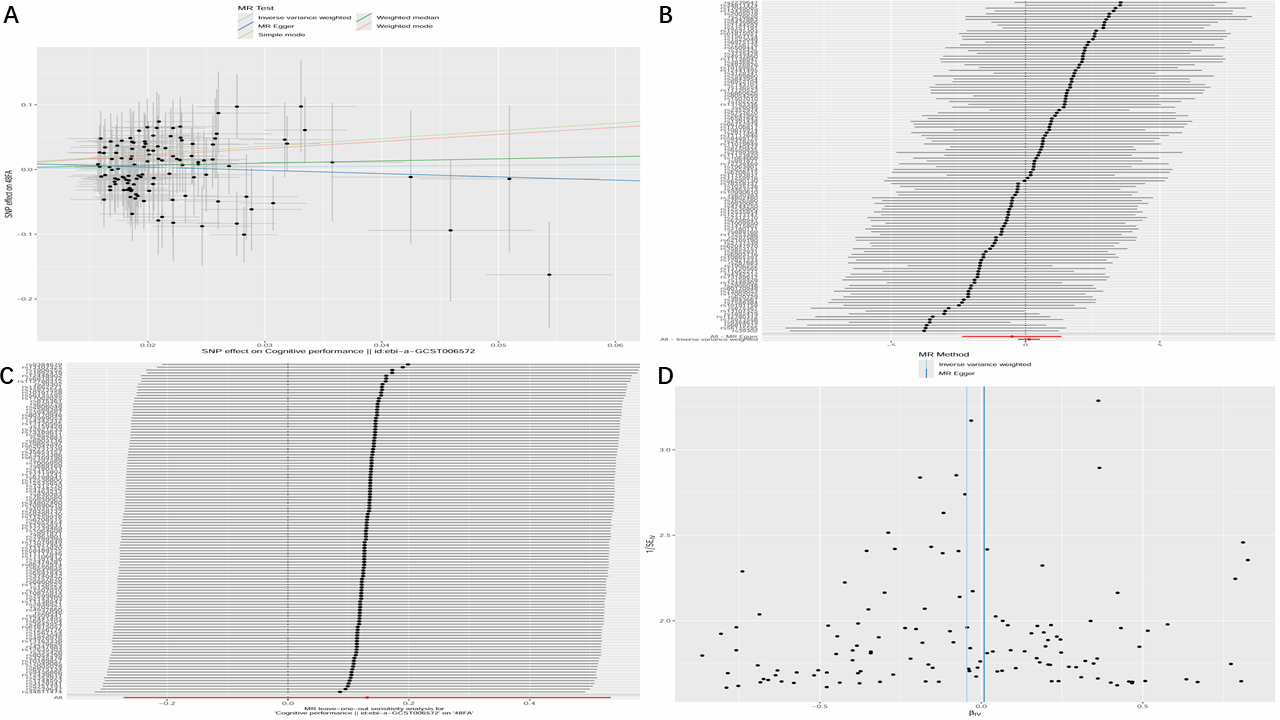


**Supplementary Figure 11.** Scatter plot (A), forest plot (B), and “leave-one-out” analysis (C) for MR analysis of Cognitive performance and FA, funnel plot (D).


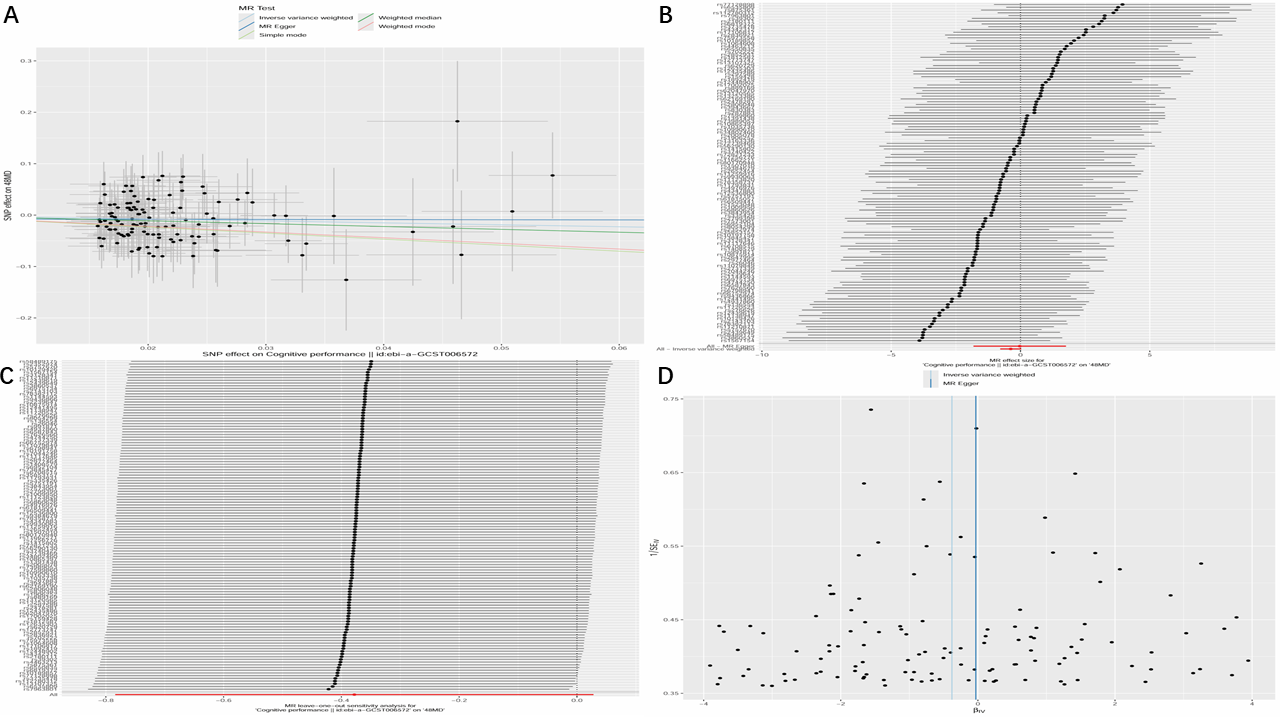


**Supplementary Figure 12.** Scatter plot (A), forest plot (B), and “leave-one-out” analysis (C) for MR analysis of Cognitive performance and MD, funnel plot (D).
